# Upconverted electroluminescence via Auger scattering of interlayer excitons in van der Waals heterostructures

**DOI:** 10.1038/s41467-019-10323-9

**Published:** 2019-05-27

**Authors:** J. Binder, J. Howarth, F. Withers, M. R. Molas, T. Taniguchi, K. Watanabe, C. Faugeras, A. Wysmolek, M. Danovich, V. I. Fal’ko, A. K. Geim, K. S. Novoselov, M. Potemski, A. Kozikov

**Affiliations:** 1 Laboratoire National des Champs Magnetiques Intenses, CNRS-UGA-UPS-INSA-EMFL, 25 Rue des Martyrs, 38042 Grenoble, France; 2 Faculty of Physics, University of Warsaw, ul. Pasteura 5, 02-093 Warsaw, Poland; 3 School of Physics and Astronomy, University of Manchester, Oxford Road, Manchester, M13 9PL UK; 4 National Graphene Institute, University of Manchester, Oxford Road, Manchester, M13 9PL UK; 5 Centre for Graphene Science, College of Engineering, Mathematics and Physical Sciences, University of Exeter, Exeter, EX4 4QF UK; 6 National Institute for Materials Science, 1-1 Namiki, Tsukuba, 305-0044 Japan; 7 Henry Royce Institute for Advanced Materials, M13 9PL Manchester, UK

**Keywords:** Materials for devices, Nanoscience and technology

## Abstract

The intriguing physics of carrier-carrier interactions, which likewise affect the operation of light emitting devices, stimulate the research on semiconductor structures at high densities of excited carriers, a limit reachable at large pumping rates or in systems with long-lived electron-hole pairs. By electrically injecting carriers into WSe_2_/MoS_2_ type-II heterostructures which are indirect in real and k-space, we establish a large population of typical optically silent interlayer excitons. Here, we reveal their emission spectra and show that the emission energy is tunable by an applied electric field. When the population is further increased by suppressing the radiative recombination rate with the introduction of an hBN spacer between WSe_2_ and MoS_2_, Auger-type and exciton-exciton annihilation processes become important. These processes are traced by the observation of an up-converted emission demonstrating that excitons gaining energy in non-radiative Auger processes can be recovered and recombine radiatively.

## Abstract (web summary)

The authors present electroluminescence measurements of light-emitting devices based on van der Waals heterostructures, and observe a lower than expected threshold voltage for intralayer electroluminescence, attributed to non-radiative Auger-type recombination of interlayer excitons and resulting energy transfer.

## Introduction

Type-II heterostructures made of semiconducting monolayer transition metal dichalcogenides (TMDs) are excellent systems to study indirect interlayer excitons and their properties, in particular at high charge carrier densities. In the regime of large carrier injection, many phenomena and processes become important that are negligible at low injection rates^1–3^. One of those processes is Auger recombination, a non-radiative decay of non-equilibrium carriers, inherent to any semiconducting material. Since for the Auger process to proceed at least three charge carriers are required, the process gains importance with increasing carrier densities. Notably, Auger-type processes become also a factor in limiting the performance of light-emitting diodes (LEDs) at high output power^4^. The importance of Auger-type exciton-exciton scattering processes was evidenced for direct intralayer excitons in semiconducting TMD monolayers by optical pump probe and time-resolved photoluminescence experiments^5–10^. Here, we study indirect interlayer excitons in electroluminescent devices with a particular focus on the high carrier density regime.

**Fig. 1:**
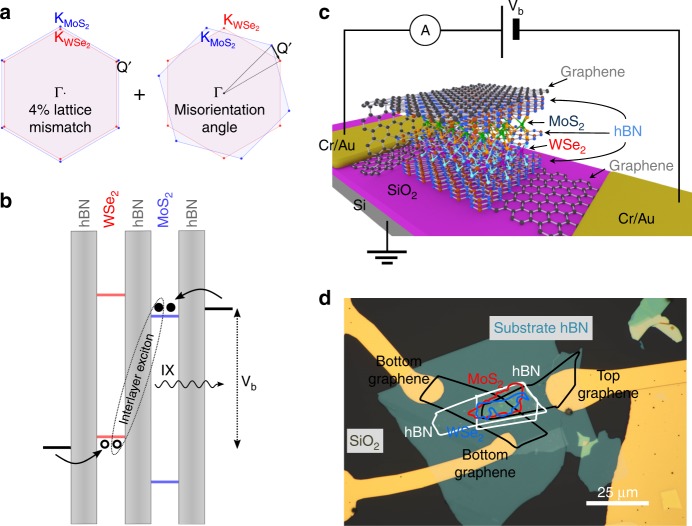
Sample structure and selective charge carrier injection. **a** Brillouin zones of WSe_2_ (red) and MoS_2_ (blue) illustrating the momentum Q’ arising due to lattice mismatch and misorientation angle. **b** Schematic illustration of the type-II band alignment for the MoS_2_/WSe_2_ heterostructures with a middle monolayer hBN spacer. The conduction and valence band of WSe_2_ (MoS_2_) are represented by red (blue) lines. The hBN layers are represented by gray-shaded rectangles. The black lines depict the quasi Fermi levels in the bottom and top graphene electrodes for an applied voltage above the threshold for hole tunneling into WSe_2_ and electron tunneling into MoS_2_. The dashed ellipse indicates the formation of an interlayer exciton consisting of an electron in the conduction band of MoS_2_ and a hole in the valence band of WSe_2_. **c** Schematic drawing of the heterostructure shown in (**b**). **d** Optical microscope image of the active area of device B1

In this work, we employ constant electric carrier injection, which is the common operating condition for LEDs, to study the Auger recombination and exciton–exciton interactions. For this approach, without strong pumping, one has to render the radiative recombination inefficient to achieve large charge carrier densities. To this end, we chose to study van der Waals (vdW) heterostructures with MoS_2_/WSe_2_, which are stable semiconducting TMDs that feature a type-II band alignment and a large lattice mismatch (~4%), see Fig. 1a, b. Such heterostructures allow us to study long-lived interlayer excitons (IX) in the energy range of 1–1.3 eV, important for silicon photonics and telecommunication, with an emission wavelength that can be tuned by an applied electric field. To even further suppress the radiative recombination across the indirect bandgap, we produced devices with a monolayer hBN spacer between the TMDs. In such devices, the Auger processes are strongly enhanced, dominating the exciton dynamics. Comparison of the behavior of samples with different spacers allows us to establish the qualitative characteristics of exciton–exciton interaction. Besides Auger processes, there is additional interest in the regime of high carrier densities motivated by recent progress in TMD-based excitonic devices^11^ and theoretical predictions that vdW heterostructures could allow observing effects like the condensation of excitons^12^ or high-temperature superfluidity^13^.

Another ingredient besides the reduction of radiative recombination needed to establish large IX populations is the possibility to selectively inject electrons only into one material and holes only into the other. We met this prerequisite by using tunneling injection through graphene electrodes in vertical heterostructures. In contrary to optical injection^11,14–19^, for which electrons and holes are almost exclusively created in the same and not in different materials, the electrical injection scheme circumvents the competing short-lived direct intralayer recombination, since no charge carriers of different types are present in the same material. The injection process is illustrated in Fig. 1b, and electroluminescence (EL) becomes the method of choice to characterize the devices. Strikingly, besides the observation of the EL of the IX, we observe a large upconversion effect enabling intralayer emission of WSe_2_ and MoS_2_ at voltages well below the voltages corresponding to the respective excitonic bandgaps.

## Results

### Van der Waals heterostructures for interlayer excitons

A schematic drawing of a typical device is presented in Fig. 1c. Si/SiO_2_ is used as the substrate for the vdW heterostructure with the following layer sequence: Gr/3–5 hBN/1 WSe_2_/0–1 hBN/1 MoS_2_/3–5 hBN/Gr. For all devices discussed in the article, the outer hBN barriers were three-to-five-layer thick. The middle thin hBN spacer was either one layer thick or completely absent. Based on the presence of this hBN spacer, we can divide the devices studied into two groups: with and without a monolayer hBN spacer separating the TMDs. Our study comprised seven devices, out of which five were fabricated with and two without a monolayer hBN spacer. The devices with hBN spacer are numbered A1–A5 and samples without hBN spacer B1, B2. For all these devices, the two TMDs were aligned under the optical microscope (estimated accuracy 2°). An optical microscope image of such a sample is shown in Fig. 1d. Details about the device fabrication and the basic optical properties can be found in ref. ^20^ and in the Methods section. Figure 1b illustrates the type-II band alignment of a WSe_2_/MoS_2_ vdW heterostructure with a monolayer hBN spacer and the tunneling pathways for charge carriers upon application of a voltage between the top and bottom graphene electrodes. The reported values for the band offsets of monolayer WSe_2_ and MoS_2_ are in the range of ~0.6–0.7 eV^21–23^ for the conduction band offset, between ~0.8 and 1.1 eV^21,22,24^ for the valence band offset and in the range of ~0.9–1.3 eV^21–23^ for the interlayer bandgap. For the case depicted in the sketch, the voltage is large enough to enable electron injection into the MoS_2_ conduction band and hole injection into the WSe_2_ valence band (V_b_ > 1 V), but the voltage is below the threshold for direct WSe_2_ and MoS_2_ electron–hole injection (V_b_ < 1.7 and 1.9 V, respectively)^25^. This selective injection of a given carrier type into only one material together with the large band offsets results in an extremely large charge build up at the interface and hence facilitates large IX populations.

### Tunable electroluminescence of interlayer excitons

**Fig. 2:**
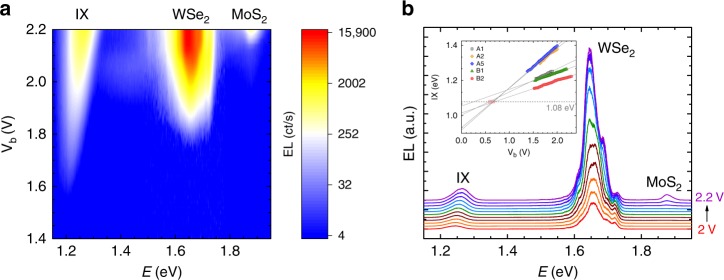
Interlayer excitons. **a** False color contour plot of the EL spectra as a function of bias voltage for sample B1 without monolayer hBN spacer. **b** EL spectra for biases in the range of V_b_ = 2.0–2.2 V extracted from (**a**). The spectra are vertically shifted for clarity. The inset in panel (**b**) shows the peak position of the IX as a function of bias for five different samples and linear fits to the dependencies. The gray dashed line marks the energy of 1.08 eV, which is an estimation for the energy of a presumptive IX without electrically injected carriers. We obtain this value by using threshold voltages of ~0.6–0.7 V (red shaded area) for measurable carrier injection extracted from photoluminescence and reflectance contrast measurements (see Supplementary Note 4)

First, we discuss the experimental results typical for the devices (B1 and B2) without hBN spacer between the TMDs. Figure 2a shows the evolution of the EL as a function of bias voltage in a broad energy range for sample B1. The EL spectra, Fig. 2b, show three contributions: an emission originating from MoS_2_ at around 1.9 eV, a broad band at 1.7 eV related to WSe_2_ and a third peak at around 1.2–1.3 eV, which we attribute to IX emission. We can pinpoint this peak to originate from IX since we observe a strong blueshift as a function of bias voltage. This blueshift is a consequence of the electric field that builds up in our vertical tunneling structure upon applying the bias voltage, which results in an increase of the distance between the conduction band edge of MoS_2_ and the valence band edge of WSe_2_ (see Fig. 1b). This behavior was universal for all devices studied showing linear shifts with slopes in the range of 90–200 meV/V (see Fig. 2b). The different slopes are the result of the different effective thicknesses of the barriers. The shift of the IX is a measure of the electric field and can be used to estimate the IX density (see Supplementary Note 4). To determine the total IX shift, one has to estimate the energy of a presumptive IX without electrically injected carriers. To this end, photoluminescence and reflectance contrast measurements (see Supplementary Note 4) were used to extract the threshold voltages for carrier injection (red shaded area in Fig. 2b). The extrapolation of the linear fits to this voltage yields an energy of about 1.08 eV for a presumptive IX without electrically injected carriers, in good agreement with an interface bandgap of 1.08 eV, recently extracted from transport measurements^23^. The IX emission energy in vertical vdW heterostructures is easily tunable, which is an interesting feature for many prospective applications. In contrast, no significant shift of the intralayer transitions as a function of bias voltage can be observed, in agreement with the fact that transitions in the same material are not sensitive to relative band movements caused by the electric field.

For both devices without an hBN spacer (results for device B2 are shown in Supplementary Note 3), the different contributions emerge one after another with increasing bias voltage. The IX emission appears first (V_b_ ~ 1.5 V), as the difference between the conduction band of MoS_2_ and the valence band of WSe_2_ constitutes the lowest barrier for electron–hole injection (see Fig. 1b). At larger voltages (V_b_ ~ 1.75 V) the WSe_2_ emission emerges, which is a result of tunneling into the intralayer excitonic states. Finally, at (V_b_ ~ 2.1 V), MoS_2_ emission becomes observable in accordance to the larger bandgap. It is interesting to note that the voltages for which EL can be observed correspond to the exciton emission energy rather than to the single particle bandgap in agreement to what has been observed for vdW heterostructures with a single WSe_2_ monolayer^25^.

### Upconverted emission of intralayer excitons

**Fig. 3:**
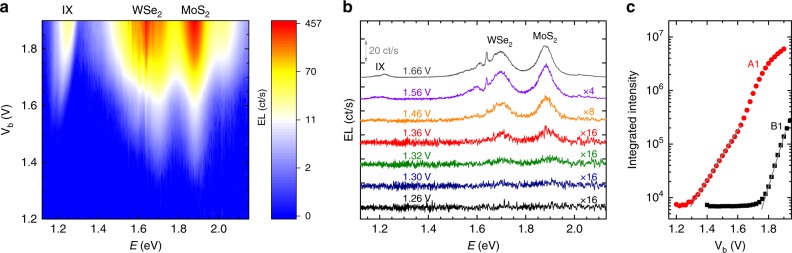
Upconverted electroluminescence. **a** False color contour plot of the EL spectra as a function of bias voltage for sample A1 with a monolayer hBN spacer. **b** EL spectra for seven different bias voltages extracted from (**a**). The spectra are vertically shifted for clarity. For a voltage of V_b_ = 1.32 V emission at energies up to around 1.9 eV are observed, clearly illustrating the large upconversion effect. **c** Comparison of the integrated EL intensity in the spectral range of intralayer emission (1.32–2.37 eV) as a function of bias voltage for sample A1 (red circles) and B1 (black squares). A background signal from the response at V_b_ = 0 V was subtracted for each spectrum. The integrated EL intensity at voltages below the onset of observable emission corresponds to the noise level of our setup of around 2 ct/s per pixel (integrated over about 4000 pixels)

The situation is different for the devices with a monolayer hBN spacer for which one expects a further reduced radiative recombination. The results are exemplary shown for device A1 in Fig. 3a (data for other devices is presented in Supplementary Note 2). Strikingly, one observes emission from both MoS_2_ and WSe_2_ at bias voltages as low as 1.3 V. The emitted photons at that voltage have an energy of around 1.7 eV (WSe_2_) and 1.9 eV (MoS_2_), which constitutes a remarkable upconversion of ~0.6 eV. These measurements reveal electrically driven upconversion for light-emitting devices based on vdW heterostructures.

An additional interesting observation is that the onset of emission is virtually the same for both MoS_2_ and WSe_2_. Moreover, we note that the emission from WSe_2_ and MoS_2_ emerges at lower applied bias voltages than the emission from the IX in contrast to samples without an hBN spacer (compare to Fig. 2a). The EL spectra presented in Fig. 3b also clearly show that for the sample with an hBN spacer, the contribution of the intralayer excitonic emission of WSe_2_ and MoS_2_ is much stronger than the IX emission. These major observations allow drawing the following important conclusions. First, the process seems not to be dependent on the upconversion energy, since it shows equal onsets and intensities for both WSe_2_ and MoS_2_. Second, the upconversion process must be almost equally probable for electrons and holes, since in order to observe these intralayer excitons one has to lift holes into the valence band of MoS_2_ and electrons into the conduction band of WSe_2_ (compare Fig. 1b). Third, although the IX has a lower emission energy of 1.2–1.3 eV compared with around 1.9 eV (1.7 eV) for the intralayer excitons of MoS_2_ (WSe_2_), these intralayer excitons emerge at lower applied bias voltages in the EL spectra. Energetic upconversion was reported in literature for different inorganic semiconducting^26–30^, as well as organic^1–3^ materials. In the case of upconversion beyond the energy-scale of phonons commonly Auger processes were identified to be responsible for the effects. The observation of substantially different onset voltages for measurable intralayer emission in samples A1 and B1 is highlighted in Fig. 3c (see also Supplementary Fig. 8). Clearly, the intralayer emission appears in our spectra at bias voltages as low as V_b_ ~ 1.3 V for sample A1, whereas this emission becomes visible at larger voltages V_b_ ~ 1.75 V in sample B1. One must, however, note that the definition of these onset voltages is somewhat arbitrary, defined by the actual experimental conditions/sensitivity (the background signal). As discussed below and illustrated in Fig. 3c, one should not expect a complete disappearance of Auger processes in sample B1, but their efficiency being orders of magnitude smaller in sample B1 as compared with sample A1.

### Auger processes and upconversion mechanism

Thanks to the purely electrical carrier injection in our devices, we can rule out nonlinear effects involving photon absorption. It is therefore intuitive to investigate whether Auger processes can account for the above-described observations. Such an Auger process would proceed in the following manner: (i) An electron in MoS_2_ and a hole in WSe_2_ form an IX that recombines non-radiatively. (ii) The excess energy is transferred to another electron in the MoS_2_ conduction band. (iii) Since the transferred energy is larger than the band offset, the electron can tunnel into the WSe_2_ conduction band. (iv) Due to the large number of holes present in the valence band of WSe_2_, the electron can form a WSe_2_ intralayer exciton and recombine radiatively, which gives rise to the characteristic light emission for WSe_2_ monolayers. A similar process would be possible for holes in the valence band of WSe_2_. With such a process, one can account for the energy independence of the process, which is true as long as the energy of the indirect transition is larger than the band offsets. However, the second aspect identified above, i.e., same probability for the upconversion of electrons and holes, is hard to imagine in such a case. For such an Auger process to be efficient, there should be a state to which the charge carrier can be excited. Yet in the case of WSe_2_, there are no bands with an energy distance of the order of the IX energy in the band structure around the K point to which a hole could be excited^31^. Even if one includes larger momenta, it is hard to imagine that such a process would be equally probable as compared with electrons for which such bands should be present. In this picture, it is also difficult to explain why upconversion sets in at lower-bias voltages than the IX emission. In order to overcome this conceptual discrepancies, we propose an excitonic Auger process instead of the above described single particle considerations in agreement with the exciton–exciton interactions observed in optical pump–probe experiments^5,7–10^.

**Fig. 4:**
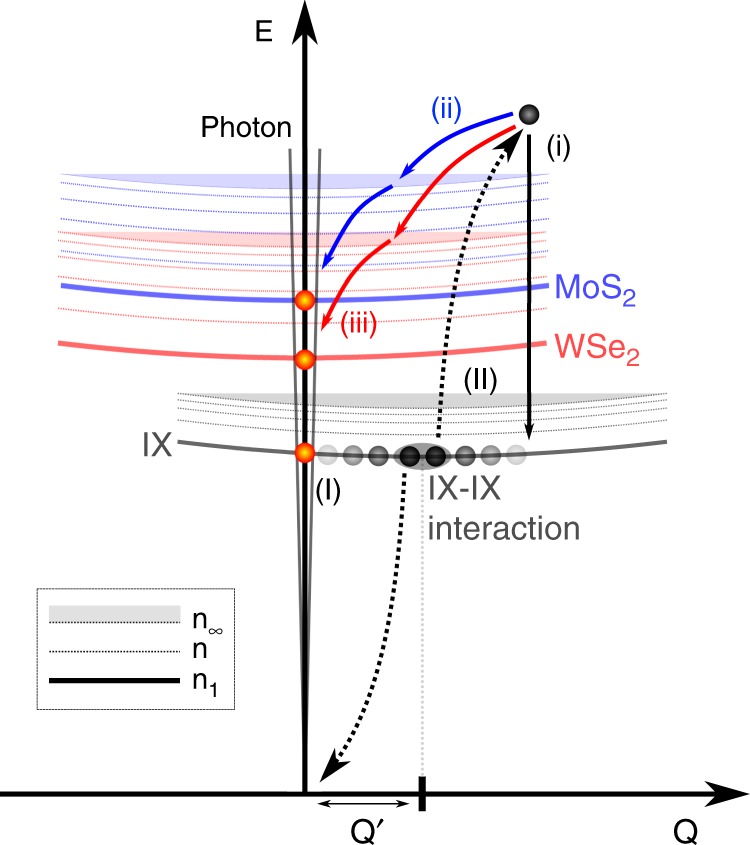
Mechanism of upconverted emission in the two-particle picture. The solid lines represent the excitonic ground-state dispersion n_1_ of the IX (gray), MoS_2_ (blue) and WSe_2_ (red). The circles stand for excitons. Q′ is the momentum mismatch as defined in Fig. 1a. The dashed lines indicate excited states n and the shaded area marks the excitonic continuum n_∞_. The gray-scale shading of circles schematically pictures the momentum distribution of excitons. A bright shading indicates less excitons for a given momentum than a dark shading. The photon dispersion is overlaid (gray lines) to mark the region of effective radiative recombination (orange circles). For the situation depicted, the bias voltage is below the threshold for direct intralayer charge injection. Mechanism (I) illustrates radiative IX emission facilitated by an increasing number of IX with large momenta. Mechanism (II) depicts excitonic Auger processes. The gray ellipse schematically highlights the interaction between two exemplary excitons. As a result of the interaction, one exciton recombines non-radiatively and transfers the energy to the other exciton (arrows with dotted lines). Relaxation: (i) describes relaxation back to the IX ground state (exciton–exciton annihilation), (ii) and (iii) relaxation to MoS_2_ and WSe_2_, respectively, which leads to upconverted intralayer emission

Figure 4 presents the proposed mechanism responsible for the upconverted EL in the two-particle picture. The gray (red, blue) solid parabola represents the exciton dispersion of the IX (WSe_2_, MoS_2_). The dashed lines represent exited states and the shaded filled area stands for the excitonic continuum. Due to the lattice mismatch, the parabola describing the IX is shifted toward higher momenta. If there is an additional misalignment angle, this will further increase the distance Q’ (see Fig. 1a). As a result of the effective selective injection of carriers, the population of IX increases. The distribution function of the population is schematically depicted by the shading of the gray circles. The orange circles stand for excitons that possess almost zero center of mass velocity and hence can recombine radiatively (light cones).

In this picture, the IX have to compensate for the additional momentum Q’ first in order to recombine radiatively. This can be achieved in two ways: (I) by increasing the IX population at larger voltages. Since with increasing bias electrons and holes that form the IX possess larger and larger momenta, a significant amount of the IX population will as well extend more and more toward larger momenta. The momentum range of efficient radiative recombination is determined by the photon dispersion, as indicated in Fig. 4. If their momentum roughly equals Q’, the IX can recombine radiatively. Otherwise, the IX remains optically silent and gives rise to an increasing population that enables mechanism (II) through IX–IX collisions. As a result, one IX recombines non-radiatively and transfers the energy and momentum to a second IX (dotted arrows in Fig. 4). This IX is excited into the continuum of excitonic states and has now three different decay channels: (i) the IX relaxes back into the IX ground state. This scenario is also called exciton–exciton annihilation. (ii) corresponds to the case where the now almost delocalized exciton relaxes into a state that corresponds to the MoS_2_ intralayer exciton. Please note that the relaxation process shown here is indirect in real space, i.e., that in the single-particle picture this would correspond to a tunneling of the electron into the MoS_2_ conduction band. Similarly possible is (iii) for which the excited exciton relaxes to a WSe_2_ exciton state. For both (ii) and (iii), the relaxation continues via intralayer excitonic states until the exciton reaches the bottom of the dispersion relation, where it can recombine radiatively.

Within this picture, we can imagine (ii) and (iii) to be equally probable, since there is a plethora of excited excitonic states for both materials, in contrast to the lack of higher energetic hole bands around the K point in the single-particle picture. One can also explain the lower voltages for the onset of upconversion compared with the onset for the IX emission. In order to recombine radiatively, the IX has to compensate for the momentum Q’ (I), but below this threshold there is already a significant population of optically silent IX present that can give rise to the upconversion (II). This difference in onsets should strongly depend on the misorientation angle that adds to the momentum Q’ due to lattice mismatch. Based on literature values for the effective masses and lattice parameters^32^ and by using an estimation of the momentum mismatch of $0.04 \cdot \overline {{\mathrm{\Gamma }}K}$ for zero misalignment and $0.1 \cdot \overline {{\mathrm{\Gamma }}K}$ for 5° misalignment^33^, we can estimate the additional kinetic energy needed to recombine radiatively to be ~10 and ~60 meV, respectively. These energies indicate that such an emission could be temperature activated, but depends sensitively on the misalignment angle. It has been shown theoretically that for such structures one should expect six light cones irrespective of the layer misalignment angle, which are situated at a nonzero center of mass velocity^33^. Consequently, the IX has to possess a large kinetic momentum or has to be scattered by defects or phonons into the light cones to recombine radiatively. Otherwise these IX remain dark as shown in Fig. 4. This fact renders the observation of PL of the IX very ineffective^18,19,23^, but on the other hand offers an ideal platform to study many-body interactions allowing more easily reaching the large IX population regime. In the more widely studied system of MoSe_2_/WSe_2_, the lattice mismatch is small (<0.1%) which makes the PL of IX observable allowing to study the properties of the IX more directly^14–17,34^, but consequently not allowing for large IX populations. For our samples measured at low temperatures, no PL of the IX could be observed in agreement with other recent reports^18,19,23^ even with additional applied voltages. Please note that we only consider *K* − *K* interlayer excitons where the electron and hole stem from the K points of the respective TMD layers. Recently, ${\mathrm{\Gamma }} - K$ interlayer excitons were observed and identified in the photoluminescence of MoS_2_/WSe_2_ vdW heterostructures^19^. These ${\mathrm{\Gamma }} - K$ interlayer excitons appear at larger energies of about 1.6 eV, which agrees with other reports of interlayer excitons in this material system^11,35,36^. In this work, we were able to reveal the emission spectra of *K* − *K* interlayer excitons which are not observable in PL. This observation is enabled thanks to our selective electrical injection mechanism, leading to large IX populations.

## Discussion

Out of the seven devices studied, three showed upconverted EL emission. An additional device showed similar behavior—e.g., intralayer emission before IX—but at larger voltages, suggesting that not the whole applied voltage dropped across the active region of the vdW heterostructure (see more detailed information on every device in Supplementary Note 1). All of these devices feature a monolayer hBN spacer. The two devices without hBN spacer did not show upconversion, as well as one sample with hBN spacer for which, however, charge injection was low and asymmetric. In our picture, we can also account for the behavior of the devices that do not show upconversion. Here, the consecutive appearance of different contributions with increasing bias voltage (compare Fig. 2a) is reflected by the increasing energetic positions of bright excitons around zero momentum (see Fig. 4) into which the charge carriers can tunnel directly at larger voltages.

Our results suggest that the probability for Auger processes and upconversion versus radiative IX recombination can be effectively tuned by changing the thickness of the middle hBN barrier. The presence of an hBN spacer between the TMDs strongly influences the radiative recombination rate for IX emission in the vicinity of the light cone. By introducing an hBN spacer, we increase the distance between the electron and the hole and consequently decrease the wave-function overlap. We can therefore expect the radiative recombination rate to be quenched in the case of devices from group A. Indeed, we observe that the upconverted intralayer emission is more intense than the IX emission (see Fig. 3). Devices with an hBN spacer hence favor larger IX densities, since the radiative IX recombination at almost zero momentum becomes less effective.

To experimentally confirm this conjecture, we estimated the IX density with two different methods: (i) by analyzing the apparent evolution of the exciton versus trion resonances in the MoS_2_ layer in photoluminescence (PL) and reflectance contrast (RC) measured as a function of bias voltage and (ii) by applying a simple capacitor model to account for the observed blueshift of the IX peak in the EL measured as a function of applied bias voltage, thus deducing the built-in electron–hole charge. Both methods (see Supplementary Note 4 for details on measurements and analysis) yield IX densities in the range of 10^12^ cm^−2^ for sample A1, but distinctly smaller densities, in the range of 10^11^ cm^−2^, for sample B1. With these findings, we indeed confirm our expectations that a larger accumulation of interlayer excitons appears in samples with an hBN spacer, favoring the observation of Auger processes in such structures.

However, the presence or absence (strong or weak efficiency) of the Auger processes should also be discussed with respect to an apparent ratio d/a_B_ of the inter exciton distance d to the exciton Bohr radius a_B_. With *n* = 3.7 · 10^12^ cm^−2^ for sample A1 and *n* = 6.1 · 10^11^ cm^−2^ for sample B1 (see Supplementary Note 4), we obtain ${\mathrm{d}}_{{\mathrm{A}}1} = 2/\sqrt {\pi n} \sim 5.9\,{\mathrm{nm}}$ and d_B1_ ~ 14.4 nm, respectively for samples A1 and B1. To estimate a_B_, we note that the Bohr radius of an interlayer exciton is expected to be about a factor of two larger than that of the intralayer exciton^37^. The exciton Bohr radius for intralayer excitons of semiconducting monolayer TMDs is known to be sensitive to the dielectric surrounding and varies in the range of 1–1.7 nm^38,39^. Thus, given their hBN encapsulation, we roughly estimate a_B_ ~ 3 nm for the interlayer exciton Bohr radius in our structures and conclude that d_A1_/a_B_ ~ 2 for sample A1 and a significantly larger value d_B1_/a_B_ ~ 5 for sample B1. The conditions met by sample A1 are close to the Mott transition for interlayer excitons, which enables the observation of many-body effects in this case^39^. In addition, one may speculate that d/a_B_ can be further reduced in sample A1, as the introduction of an hBN spacer could lead to the reduction of the binding energy of the intralayer exciton in sample A1, as compared with sample B1. However, this reduction was calculated to be rather small^37^, thus charge accumulation seems to be a decisive factor for the observation of the Auger-type processes in our samples.

An expected, super-linear (ideally quadratic) behavior of the intensity of the Auger upconverted emission as a function of the IX density is another issue to be considered. It is tempting to trace this behavior by investigating the dependence of the emission intensity versus driving current. However, this dependence may also be super-linear due to trivial effects not related to the Auger process, like leakage currents due to device imperfections or exciton-trapping centers, which are also observed in III–V quantum well structures^40^. For most of our devices, we obtain an overestimation of the super-linear behavior of emission intensity as a function of driving current, due to current contributions that are not related to the emission process. Instead, for device A1, showing excellent I–V characteristics, the extracted super-linear trend in the regime of the Auger upconverted emission appears reliable, since in the case of this device the trend becomes practically linear in the direct intralayer injection regime, when the applied voltages exceed the excitonic bandgaps of the TMD monolayers (see Supplementary Fig. 9). It is clear that more work on the role and nature of defects and a better control of the interfaces in vdW heterostructures is needed to tackle this issue for all devices.

In conclusion, we studied Auger processes in especially chosen type-II vdW heterostructures that enable large populations of optically silent IX. The double indirect nature of these excitons allows recovering a part of the excited Auger carriers through relaxation in optically active states. These states lie higher in energy than the initial IX, which results in the emission of upconverted photons. The large IX populations established thanks to the purely electrical tunnel injection allowed us to observe EL of otherwise silent IX, which shifts due to the electric field. It is shown that the IX energy can be tuned by up to 200 meV/V. A purely excitonic Auger process is proposed that, in contrary to Auger processes based on the single-particle picture, accounts for the major characteristics of the EL as a function of bias voltage. The results suggest that the efficiency of the upconversion mechanism depends on the hBN layer between the active TMDs. The revealed variable nature of the IX–IX interactions is of key importance for future TMD-based optoelectronic device engineering and is important for any attempt aiming toward exciton condensation or superfluidity in such structures.

## Methods

### Device fabrication

All devices were fabricated on doped silicon substrates covered with silicon oxide 90 -nm thick. Top graphene flakes were exfoliated on a silicon substrate spin coated with PMGI (polymethylglutarimide) and PMMA (polymethyl methacrylate). After dissolving PMGI, PMMA membranes with graphene were used to pick up other flakes. MoS_2_ and WSe_2_ were exfoliated on a silicon oxide substrate (290 -nm thick) spin coated with PPC (polypropylene carbonate). Thin boron nitride and bottom graphene were exfoliated on silicon oxide substrates 70- and 90 -nm thick, respectively. In some devices, we used substrate hBN about 30 -nm thick exfoliated on silica substrates 90 -nm thick. Light-emitting diodes were assembled by picking TMDs and hBN flakes one after another with the top graphene flake and transferring the resulting stack on top of the bottom graphene. The PMMA membrane was then dissolved in acetone. In some devices, the flakes were picked up by top graphene and peeled onto the substrate hBN. After the assembly, electron-beam lithography was used to define a mask for contacts followed by evaporation of Chromium/Gold (Cr/Au, 3/50 nm).

### Optoelectronic measurements

The optoelectronic measurements were performed using two different helium flow cryostats (Janis ST-500, Oxford Instruments MicrostatHires) and a helium bath cryostat. All the measurements shown in the main text were taken at liquid helium temperature. The signal was collected using a 0.5-m long spectrometer equipped with liquid nitrogen-cooled charge-coupled-device (CCD) camera. Electrical measurements were performed using a Keithley 2450 source-measure unit synchronized with the spectrometer. A diode type behavior was observed for all devices.

## Supplementary information

Supplementary Information

## Data Availability

The data supporting the findings of this work are available from the corresponding author upon reasonable request.
