# Supplementary material for: Upconverted electroluminescence via Auger scattering of interlayer excitons in van der Waals heterostructures
Source: Nat Commun. 2019 May 27;10:2335. doi: 10.1038/s41467-019-10323-9 (PMC6536535; doi:10.1038/s41467-019-10323-9)
Supplement: Supplementary file 1 — Supplementary Information [file 41467_2019_10323_MOESM1_ESM.pdf]

Supplementary Information

Upconverted electroluminescence via Auger scattering of interlayer excitons in van der Waals heterostructures

Johannes Binder et al.

Supplementary Note 1: Studied devices, their I-V characteristics and comments on their performance

	IV curve	hBN spacer	Upconversion	comments
A1	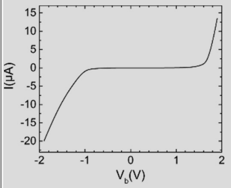	YES	YES	low leakage currents; steep onset in IV curve
A2	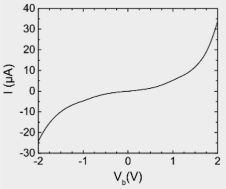	YES	YES	large leakage and total currents; no pronounced PL and EL signals from the MoS_2 monolayer
A3	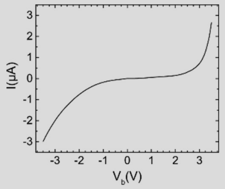	YES	YES<br>(at larger voltages)	low leakage currents; steep onset in IV curve but at larger voltages;<br>parasitic voltage drop; larger voltages needed to obtain an upconversion-like behaviour:<br>WSe_2, MoS_2 intralayer excitons emerge at lower voltages than IX
A4	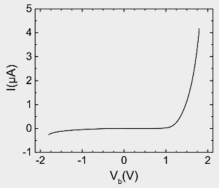	YES	YES	low leakage currents; steep onset in IV curve<br>only room temperature data; contacts ceased to work during thermal cycling
A5	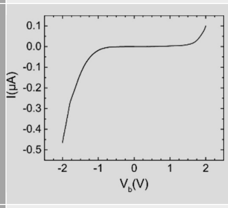	YES	NO	very low currents; PL shows asymmetric charge injection; charge injection not effective at positive bias voltages (in the range where upconverted EL is observed for other devices)
B1	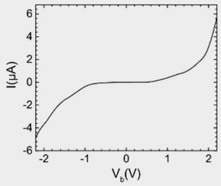	NO	NO	results are similar to sample B2;<br>good reproducibility of EL(V_b) dependence
B2	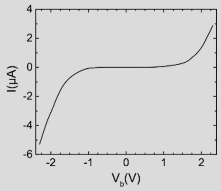	NO	NO	results are similar to sample B1;<br>good reproducibility of EL(V_b) dependence

Supplementary Figure 1. Current-voltage characteristics and additional information for all seven devices studied.

Altogether seven devices were studied (see Supplementary Figure 1). Five samples (A1, A2, .. A5) comprise an hBN spacer between the TMD monolayers, whereas the two other samples (B1 and B2) were fabricated without an hBN spacer. Each device was electrically tested and current-voltage (I-V) characteristics (see Supplementary Figure 1) were recorded. The I-V curves for all seven samples display tunneling diode operation, however the actual quality of the device performance, mostly determined by leakage currents, varies from sample to sample. Close to optimal is the device A1, with low leakage currents. Device A4 has only been tested at room temperature due to contact failure while cooling down the sample. The I-V characteristic of device A5 is quite distinct from the characteristics of the other samples. It is asymmetric and displays surprisingly low currents. Device A5 is the only sample with an hBN spacer that did not show upconverted emission. Both samples without an hBN spacer (B1 and B2) did also not show any upconverted emission. These two samples display very similar results when studied with electroluminescence (EL), but also photoluminescence (PL) and reflectance contrast (RC) experiments.

Supplementary Note 2: Upconverted emission in other devices

In this section we present the EL as a function of bias voltage for other samples that show upconverted emission. As shown in Supplementary Figure 2, sample A2 is another device which clearly shows the sub-bandgap EL. We note, however, that in this sample the emission, both in EL and PL, is only due to the WSe_2 monolayer whereas no signal associated with MoS_2 monolayer could be observed.

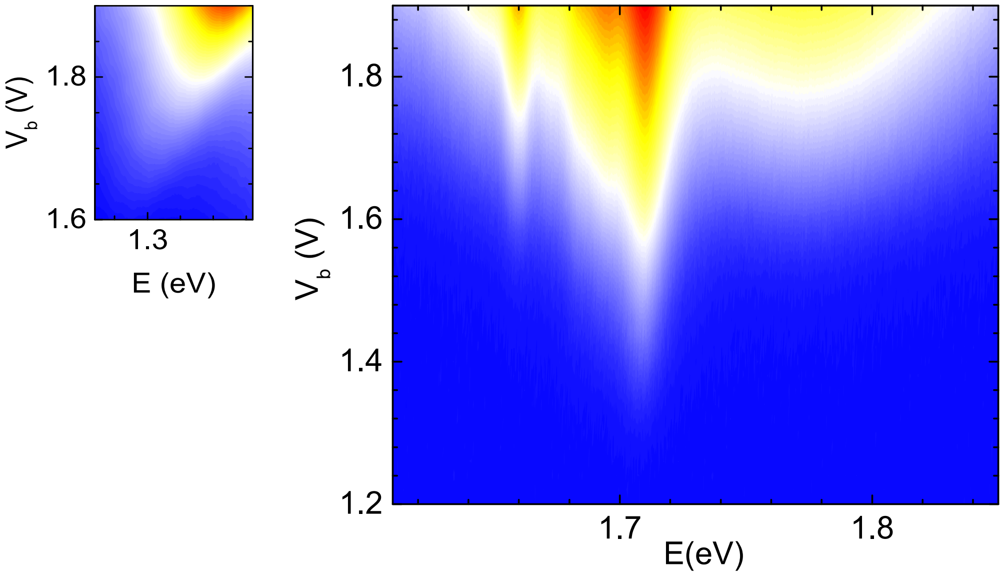

Supplementary Figure 2. Sample A2 with a monolayer hBN spacer showing the upconverted EL. For this device no intense signal from MoS_2 has been observed, both in EL and PL. The monolayer-like emission, including the upconverted emission, is mainly associated to the WSe_2 monolayer. In agreement with the results shown in the main text, the emission of the IX is emerging at larger voltages ~ 1.6 V as compared to the WSe_2 signal at ~ 1.3 V.

To clearly demonstrate the onset of the "sub-bandgap" EL, we show a series of EL spectra measured in the vicinity of the threshold voltage, both for sample A1 (from the main text) and sample A2. For device A1 we start to see EL corresponding to the A-exciton resonance of the WSe_2 and MoS_2 monolayers at voltages as low as $V_b = 1.30$ V. For device A2, even at voltages of $V_b = 1.28$ V electroluminescence of the WSe_2 monolayer can be observed.

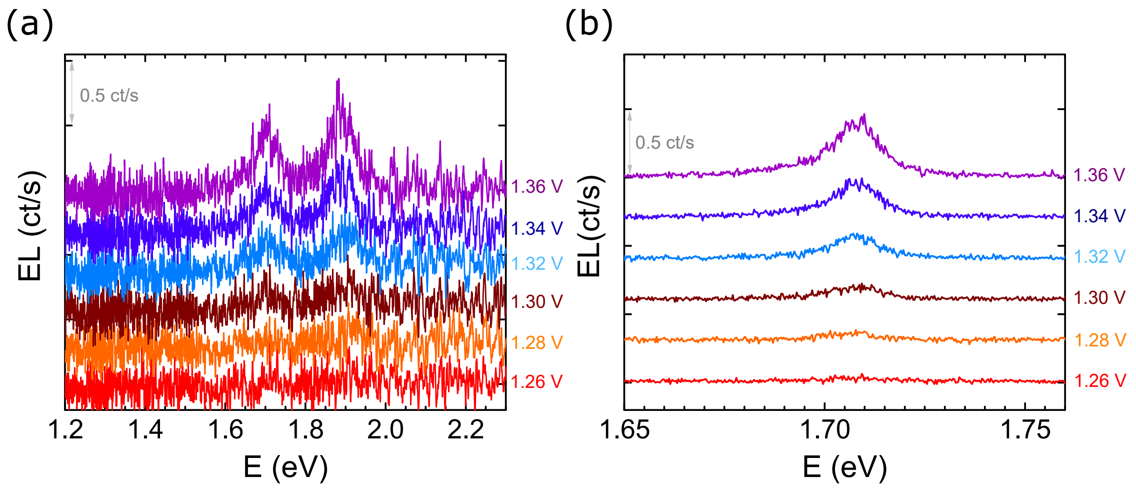

Supplementary Figure 3. Comparison of the EL spectra close to the onset of the upconverted emission for (a) sample A1 and (b) sample A2. For sample A1 (A2) EL can be observed at voltages as low as $V_b = 1.30$ V ($V_b = 1.28$ V). The spectra are shifted vertically for clarity.

The EL of device A3, shown in Supplementary Figure 4, shows similar characteristic features like device A1, discussed in the main text. The EL of the WSe_2 and MoS_2 contributions emerge at lower bias voltages than the IX emission and the onset of the emission due to the A-exciton resonance of WSe_2 and MoS_2 is virtually the same. The only major difference is that larger voltages are needed to observe emission. We believe that the larger voltages can be explained by an additional voltage drop on our device, so that the overall applied voltage does not correspond to the voltage dropping across the active area. This conclusion agrees well with the currents at larger voltages ($V_b > 3$ V) observed in the IV characteristics, which are similar to the currents for other devices biased at lower voltages ($V_b < 2$ V), see Supplementary Figure 1. Given the fact that besides the parasitic voltage drop all other characteristics are similar, we refer to this behavior as "upconversion-like". Please note that for the devices that did not show upconverted emission, (i.e., the device B1 from the main text and sample B2 discussed in the next section) the EL of the monolayer TMDs emerges according to the magnitude of the bandgap, but the IX appears first at lower bias voltages.

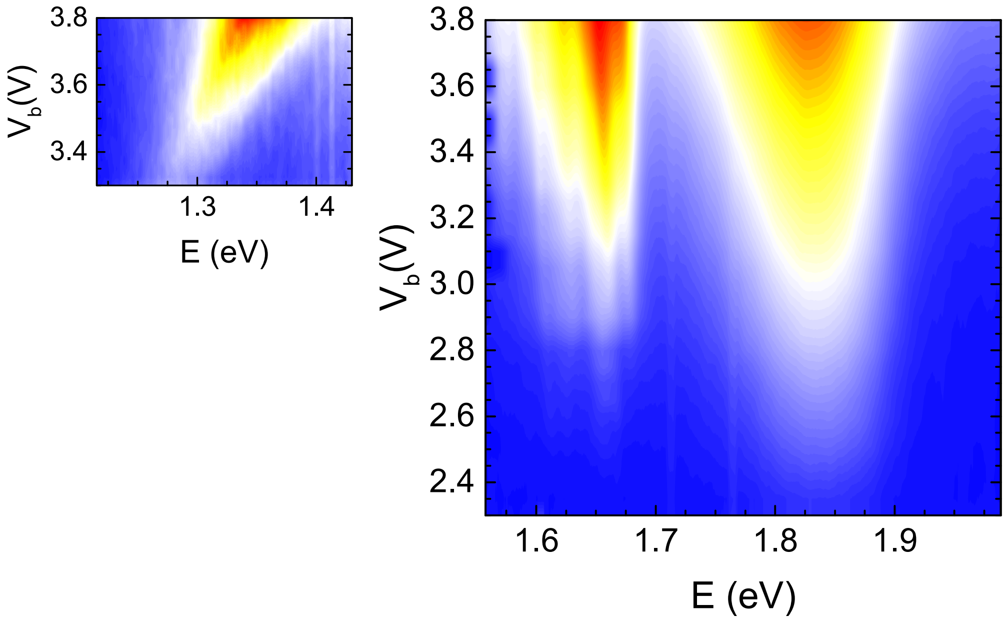

Supplementary Figure 4. Sample A3 with a monolayer hBN spacer. Similar to the other samples for which upconversion is observed the signals from WSe_2 and MoS_2 emerge at lower voltages than the emission corresponding to the IX. Most probably the voltage does not solely drop across the active area. This explains the larger voltages needed to observe the effects.

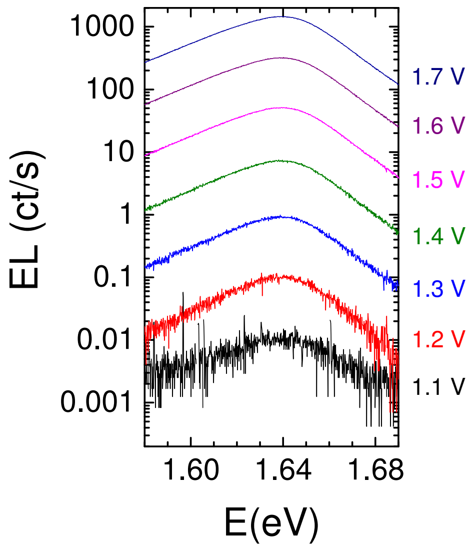

Supplementary Figure 5. Sample A4 with a monolayer hBN spacer showing upconversion. For this sample only measurements at room temperature were possible. At room temperature a broad upconverted peak corresponding to WSe_2 can be clearly seen. EL at energies around 1.64 eV is observed at bias voltages as low as 1.1 V.

Supplementary Figure 5 presents results for device A4. Unfortunately, this device could only be measured at room temperature, since the electric contacts ceased to operate during thermal cycling. The initial measurements at room temperature, however, already show a broad upconverted emission corresponding to the A-exciton resonance of WSe_2 at around 1.64 eV for voltages as low as 1.1 V. The I-V characteristics of this device show a steep onset and low leakage currents as shown in Supplementary Figure 1.

Supplementary Note 3: Electroluminescence of devices not showing upconverted emission

Device B2, with a similar design to sample B1 discussed in the main text (no hBN spacer), shows practically the same response as B1 in our experiments. The ”regular” not upconverted behavior of the EL signal in sample B2 is illustrated in Supplementary Figure 6. The IX electroluminescence of sample B2 appears at bias voltages around 1.65 V whereas the monolayer emission due to WSe_2 and MoS_2 emerges progressively at higher voltages, 1.85 V and 2.15 V, correspondingly.

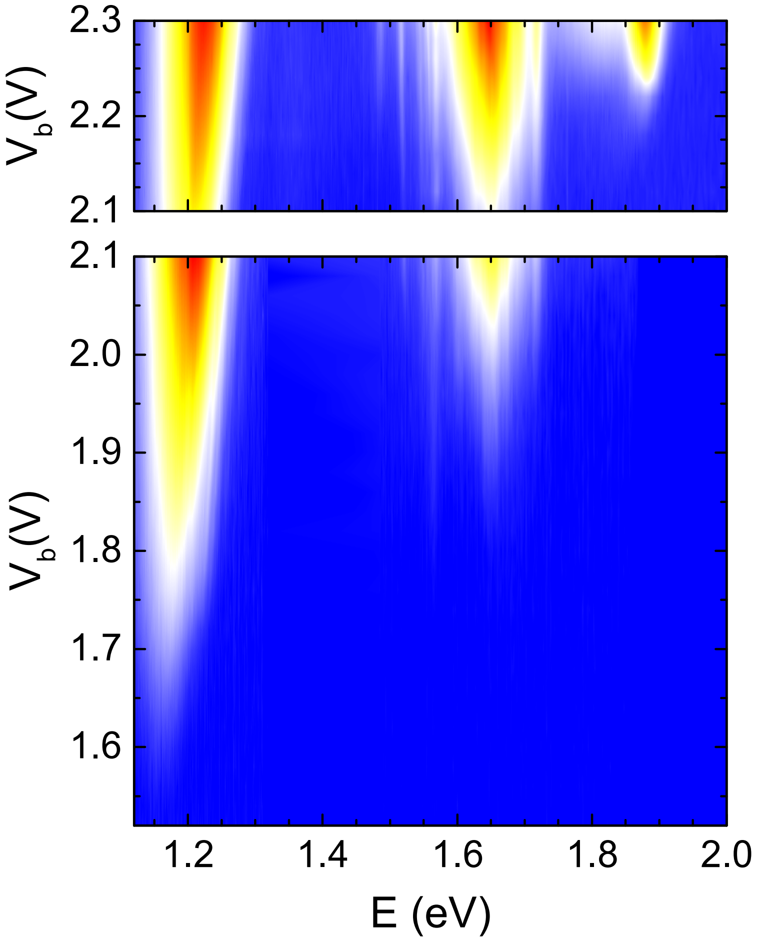

Supplementary Figure 6. Sample B2 without hBN spacer. Similar to the sample in the main text, no upconverted emission was observed. The evolution of the contributions of the IX, WSe_2 and MoS_2 is similar to that for the device B1 discussed in the main text. The two graphs show two consecutive measurements sweeps.

Supplementary Figure 7 presents the results of EL measurements for device A5. Although this device comprises a monolayer hBN spacer, no upconverted emission was observed. Device A5 is, however, very distinct from all other devices studied. It shows low overall currents and a strong asymmetry of the I-V characteristics, with larger currents in the negative voltage direction (Supplementary Figure 1). PL measurements as a function of bias voltage indicate a very asymmetric charge injection, with a trion peak emerging only in one voltage direction. These findings show that the injection through one graphene layer is much less effective and it can hence be understood that such a large difference between electron and hole concentrations does not allow to establish IX densities large enough to observe the excitonic Auger process and hence upconverted emission.

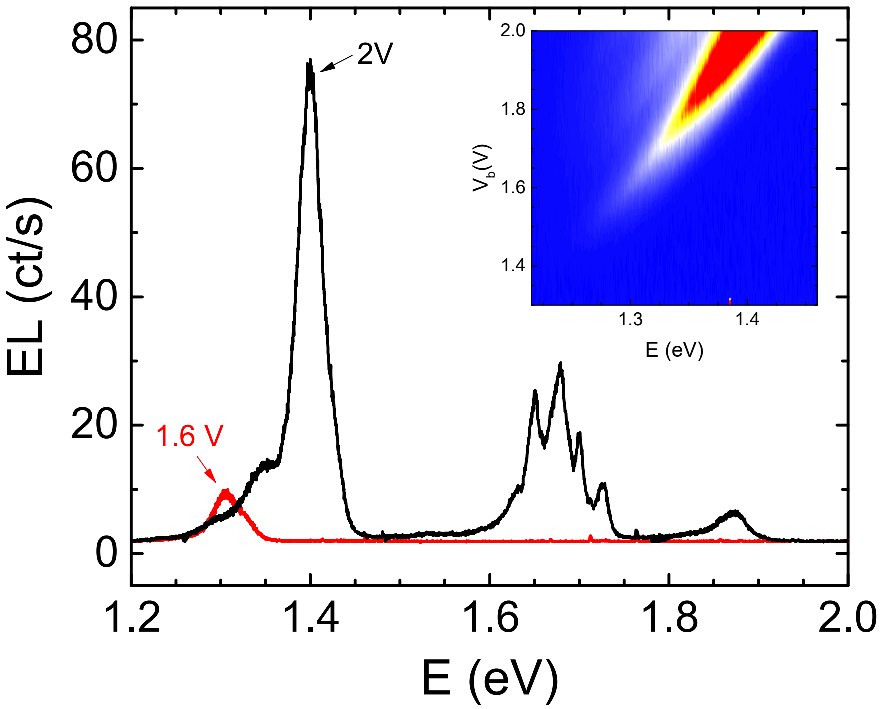

Supplementary Figure 7. Sample A5 with a monolayer hBN spacer. Two EL spectra for different bias voltages indicate that no upconverted emission was observed for this sample. In contrast to the samples showing upconverted emission, the emission from the IX emerge first. This sample showed overall low currents and PL measurements indicated a very asymmetric carrier injection. *Inset:* Map of IX emission as a function of bias voltage.

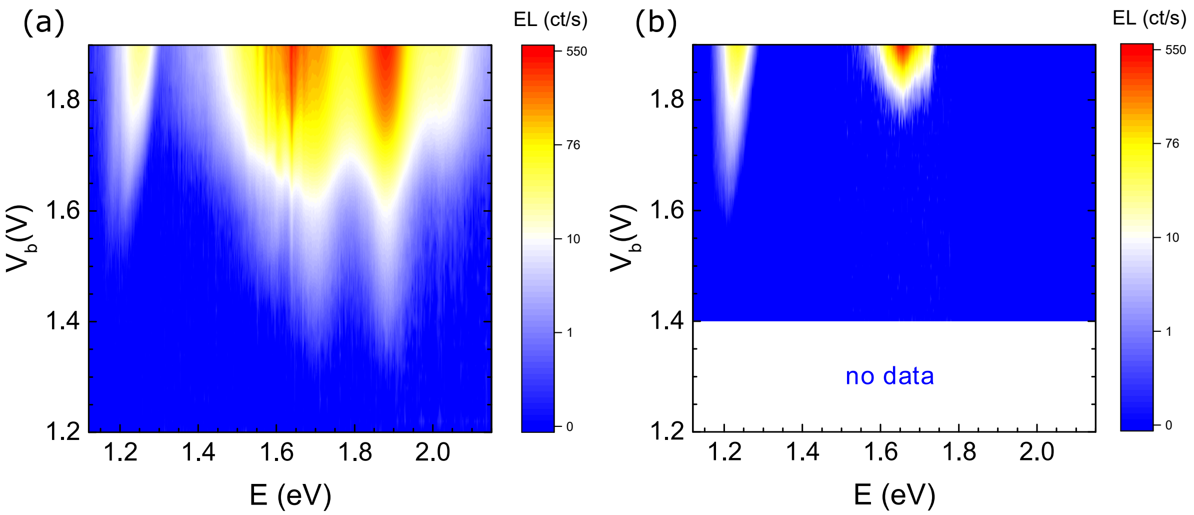

Supplementary Figure 8. Side by side comparison of the contour maps of the EL spectra measured for devices (a) A1 and (b) B1. The Supplementary Figures present the same data as shown in Figures 3 (a) and 2 (a) in the main text, but both with the same contrast (thus restricted range of bias voltages) and in the same spectral range.

Supplementary Figure 8 illustrates the different voltage onsets for intralayer emission for devices A1 and B1 on the same false color and bias voltage scale. The integrated electroluminescence intensities corresponding to these graphs were presented in Figure 3 (c) in the main text.

Supplementary Note 4: Electroluminescence, reflectance contrast and photoluminescence versus injected current/bias voltage: Supplementary data and estimation of interlayer exciton densities

1. EL intensity versus driving current

Supplementary Figure 9 presents the integrated intralayer A-exciton EL of both, MoS_2 and WSe_2, as a function of driving current for sample A1 in a log-log representation. The Figure depicts two regimes: the first regime, below a voltage of V_b=1.72 V (corresponding to the intralayer exciton emission energy of 1.72 eV), corresponds to upconverted EL. Above this voltage, direct injection into intralayer excitonic states becomes possible and we enter the second regime. Supplementary Figure 9 shows a super-linear trend for the upconverted EL. Super-linear trends are expected due to the many body nature of the process. At larger voltages, in the second regime, we observe an almost linear behavior, which agrees well with the expectations of direct charge carrier injection of intralayer excitons.

Although the extracted quantities are reasonable and within the expectations they have to be taken with care. The problem is that the driving current (x-axis of the graph) is strongly influenced by leakage currents which especially gain importance for low injection. In Supplementary Figure 9 one can observe this behavior at low currents ($I < 0.5\ \mu A$). In this regime one obtains larger exponents, which constitute an overestimation of the actual super-linear trend. The current-voltage characteristics for sample A1 (Supplementary Figure 1) show a very pronounced diode-like behavior with small currents at low voltages and a very steep onset at the threshold for direct carrier injection. However, not all the devices measured do show such an almost ideal behavior (see Supplementary Figure 1). The observed leakage leads to the above described overestimation of the super-linearity also for large voltages and currents. For device B1 for example, we can extract a dependency of $\sim I^{2.8}$ in the regime of direct carrier injection, which is in contrast to the almost linear behavior seen in this regime for sample A1, only enabled by the excellent IV characteristics of this sample. In our two-terminal devices it is very difficult to establish the actual nature of the leakage contributions, given the many possible different leakage channels for which in general one cannot assume a linear dependence on voltage. Although we observe a super-linear trend for the upconverted intralayer EL and an almost linear behavior in the direct injection

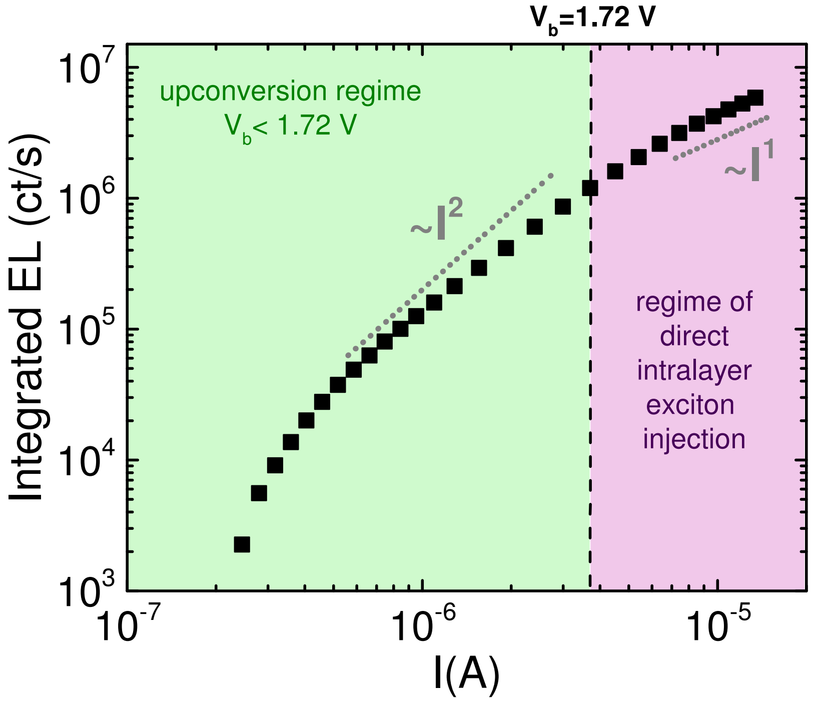

Supplementary Figure 9. Integrated EL of the intralayer exciton emission as a function of the driving current (log-log plot) for device A1. Quadratic ($\sim I^2$) and linear ($\sim I^1$) trends are presented for comparison.

regime for sample A1, an estimation relying on this measure may, however, be questionable. Optionally, we propose two other approaches to estimate the carrier concentration based on additional optoelectronic measurements.

2. *Carrier injection probed with RC measurements*

Supplementary Figure 10 presents the results of reflectance contrast (RC) measurements as a function of the applied bias voltage together with the simultaneously measured IV curve for device A1. The RC spectra were obtained by focusing a white light spot on the active area of the device followed by the subtraction of the reference spectrum of the bare SiO_2/Si substrate. In order to illustrate the changes more markedly we plot the 2nd derivative of the RC signal.

A clear change in the absorption spectrum can be observed at about 0.6 - 0.7 V. The A-exciton resonance of monolayer MoS_2 is quenched in this region and a kink can be ob-

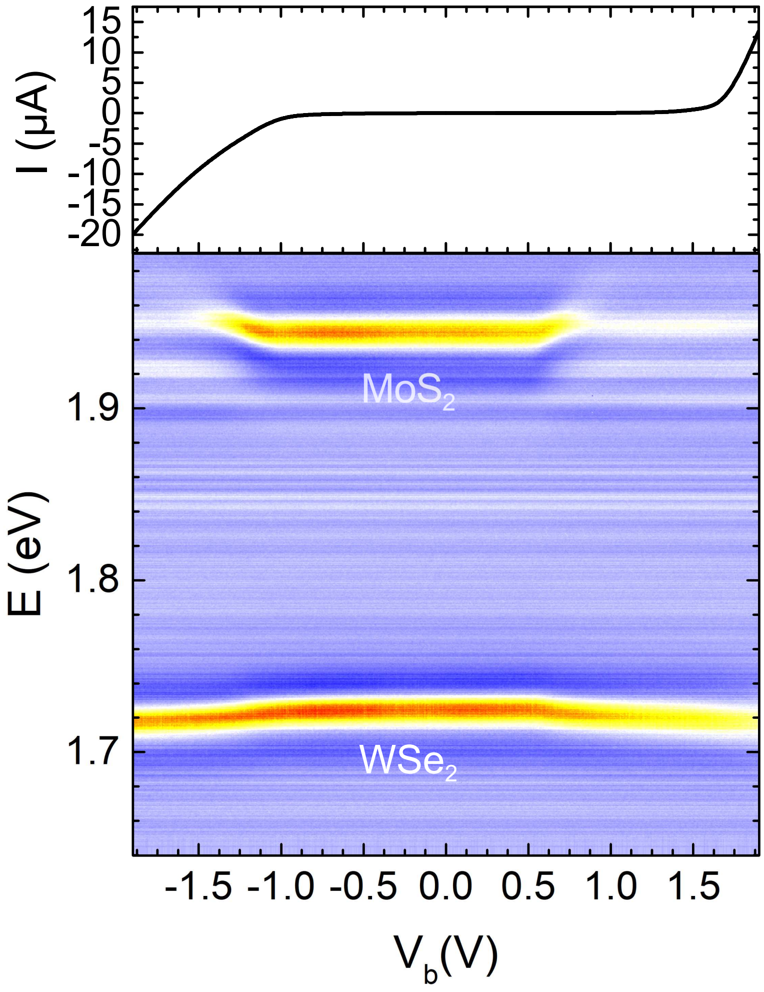

Supplementary Figure 10. Reflectance contrast measurements as a function of bias voltage for device A1. The false color map shows the 2nd derivative of the measured RC signal. The A exciton resonance could be resolved for both MoS_2 and WSe_2. A clear change in the spectra is visible at about 0.6 - 0.7 V for both monolayers. The upper panel presents the I-V characteristics recorded simultaneously with the RC data.

served for the A-exciton resonance of monolayer WSe_2. Due to the band alignment we know that electrons are injected via tunneling into the MoS_2 monolayer and holes into the WSe_2 monolayer. The quenching of the A-exciton resonance of MoS_2 shows the common behavior, for example also observed in dual-gated $MoSe_2$ monolayers[1]. The quenching of the A-exciton in the WSe_2 monolayer is somewhat less abrupt but the progressive change of its spectrum is clearly indicative of the effective injection of holes into the valence band of WSe_2. The

upper panel in Supplementary Figure 10 shows the current-voltage characteristics measured simultaneously with the the RC measurement. For negative voltages, we see a good correlation between the current onset and changes in the RC. However, in the positive direction, for which upconverted emission was observed, the current remains very small although the optical absorption measurement shows a clear change in carrier concentration. This behavior can be taken as a sign of carrier accumulation into states with a long lifetime resulting in a small current caused by carrier recombination. At voltages above 1.7 V, a direct carrier injection into intralayer excitonic states becomes possible and hence we expect a large recombination current component due to the effective radiative recombination and intense light emission in this region. The latter behavior is similar to conventional light emitting diodes.

3. PL versus bias voltage and estimation of densities of the injected carriers

Besides the RC measurements, which provide us with information about light absorption, we also performed the photoluminescence (PL) measurements as a function of bias voltage. Supplementary Figure 11 presents such a measurement for device A1.

Supplementary Figures 10 and 11 are in very good agreement. They show a quenching of the free exciton contribution at a voltage of about 0.6 - 0.7 V. This quenching allows us to conclude that at this voltage charge carriers are started to be effectively injected into states with a long lifetime yielding to a large charge build-up across the interlayer bandgap. Moreover, the PL of the MoS_2 monolayer reveals a trion resonance at voltages exceeding 0.6 - 0.7 V, which further indicates that charge carriers are injected. Supplementary Figure 12 presents a PL sweep for device B1, without hBN spacer, hence for a sample that does not show upconversion. Again a trion peak emerges, at voltages of about 0.6 - 0.7 V.

Both RC and PL measurements provide evidence for charge carrier injection at voltages well below the thresholds for intralayer exciton injection ($\sim$ 1.7 V for WSe_2) in agreement to the large expected charge build-up described in the main text. To obtain an estimation of the carrier concentration we use the dependencies presented in Ref. 2 allowing to translate the shift in energy of the trion $\Delta E = E_A - E_{A'}$, where E_A is the energy of the neutral exciton and $E_{A'}$ the energy of the trion, into a shift of the Fermi level E_f.

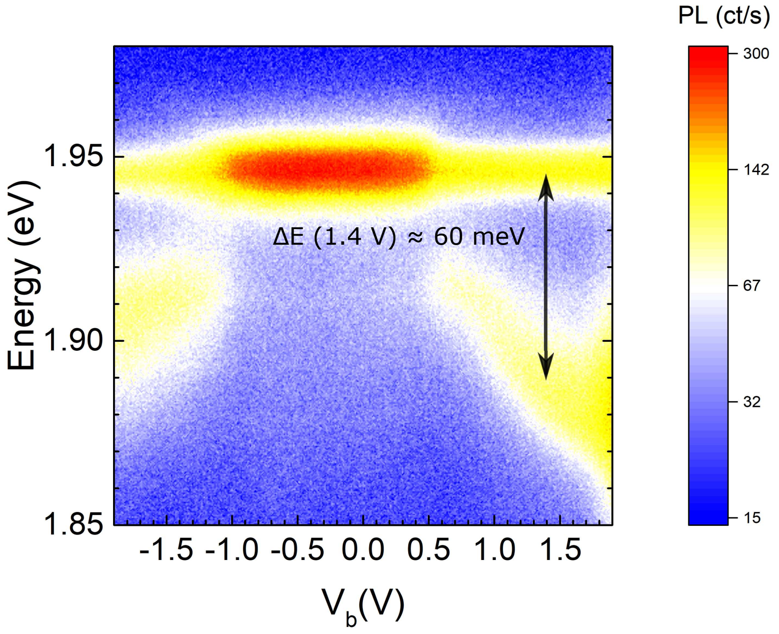

Supplementary Figure 11. Photoluminescence as a function of bias voltage for device A1 for an excitation wavelength of 514 nm. The false colour map depicts the behavior of the A-exciton resonance of MoS_2. At a voltage of about 0.6 - 0.7 V a trion peak emerges and the free excitonic emission is quenched.

$$\Delta E(V) = 1.2 \cdot E_f(V) + E_b \qquad (1)$$

where E_b is the trion binding energy. Assuming an effective mass for electrons of $m^* = 0.35\ m_e$[2] we can calculate the carrier concentration from the Fermi level with the formula:

$$n(V) = \frac{\Delta E(V) - E_b}{1.2} \cdot \frac{m^*}{\pi \hbar^2} \approx \frac{\Delta E(V) - E_b}{1.2} \cdot 1.46 \cdot 10^{14} \frac{1}{eV \cdot cm^{-2}}. \qquad (2)$$

From Supplementary Figures 11 and 12 we can extract $\Delta E_{A1} \sim 60$ meV and $\Delta E_{B1} \sim 35$ meV at a voltage of 1.4 V and a binding energy of roughly 30 meV in agreement with literature data[3,4], which yields a carrier concentration in MoS_2 of $n_{A1} \sim 3.7 \cdot 10^{12}$ cm^{-2} at a voltage of 1.4 V. A lower concentration of $n_{B1} \sim 6.1 \cdot 10^{11}$ cm^{-2} can be estimated for sample B1, without hBN spacer, not showing upconversion.

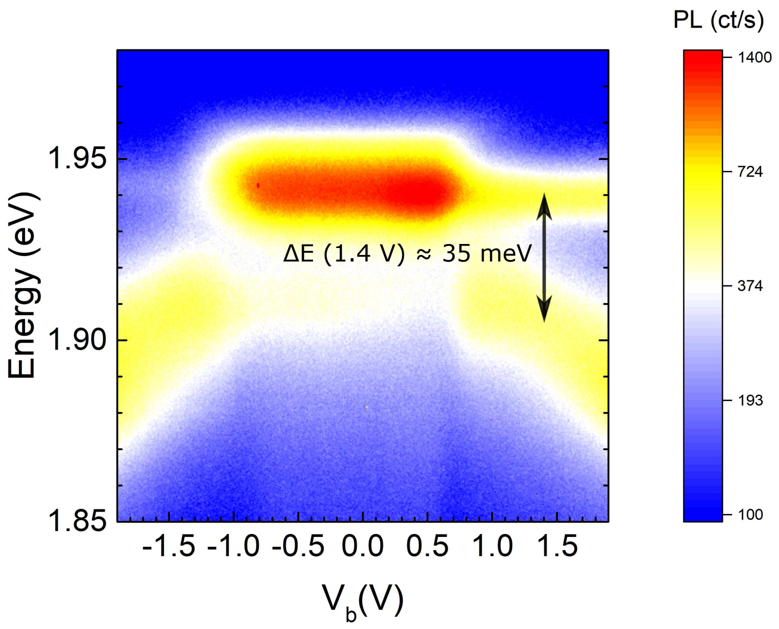

Supplementary Figure 12. Photoluminescence as a function of bias voltage for device B1 for an excitation wavelength of 514 nm. The false colour map depicts the behavior of the A-exciton resonance of MoS_2. At a voltage of about 0.6 - 0.7 V a trion peak emerges and the free excitonic emission is quenched.

4. Blueshift of the interlayer exciton with applied voltage and estimation of interlayer exciton density

The second measure allowing to estimate the carrier concentration is the blue-shift of the IX, which is a result of the relative band movements caused by an increasing electric field. The PL and RC measurements indicate that at voltages as low as 0.6 - 0.7 V charge carriers are started to be injected, hence we can conclude that at a voltage of about 1.5 V, for which the IX emerges in the spectrum, a large charge build-up must already be present at the interface between the TMDs. Based on this observation one can assume that the shift of the IX is mostly governed by the charge carriers at this interface and a simple parallel plate capacitor model can be used to the estimate a carrier concentration n:

$$n = \Delta E_{IX} \cdot \frac{\epsilon_0}{e^2} \cdot \frac{\epsilon_r}{d} \tag{3}$$

where Δ E_{IX} is the absolute blue-shift, ϵ_0 the vacuum permittivity, ϵ_r the relative permit-

tivity and d the distance between the capacitor plates. To estimate the carrier concentration we have to determine the absolute blue-shift Δ E_{IX}. We can extract the energy of the IX as a function of voltage $E_{IX}(V)$ from our EL maps revealing a linear behavior. The only unknown parameter is the energy of the IX without injected charge carriers $E_{IX}(V_0)$. As mentioned in the main text, no PL of the IX could be observed at zero voltage so we cannot directly measure this quantity. However, the PL and RC measurements provide us with an estimation concerning the minimal voltage threshold for carrier injection into the TMDs. For device A1 this would be around $V_{\text{thres}_{A1}} = 0.6$ V. We can hence use this value and extrapolate the fitted IX positions to this voltage which gives an energy of $E_{IX_{A1}}(0.6\ V) \approx 1.08$ eV. In the case of device A1 we obtain the simple formula Δ $E_{IX}(V)=E_{IX}(V)-E_{IX_{A1}}(0.6\ V)$. The general formula for all devices is

$$n(V) = (E_{IX}(V) - E_{IX}(V_{thres})) \cdot \frac{\epsilon_0}{e^2} \cdot \frac{\epsilon_r}{d} \tag{4}$$

where $E_{IX}(V_{\text{thres}})$ is the threshold voltage for charge accumulation extracted from PL.

$E_{IX}(V)$ is presented for several devices in the inset of Figure 2 in the main text. The linear trends for devices B1 and B2 have similar slopes, however the trend for device B1 is shifted to larger energies. The extrapolation for this device deviates from the others and for an extracted voltage of $V_{\text{thres}_{B1}} = 0.6$ V we obtain $E_{IX_{B1}}(0.6\ V) \approx 1.11$ eV for the presumptive energy of IX emission without injected carriers. The estimated carrier concentrations for device B2 are very similar, since due to the parallel slope, the difference $E_{IX}(V_{\text{thres}})$ is compensated by lower values of $E_{IX}(V)$. Besides the variation of ΔE_{IX} the carrier concentration will also be strongly influenced by the ratio of ϵ_r and d.

For the distance d we use values from Ref. 5 of $d_{WSe_2/hBN} = 0.528$ nm, $d_{MoS_2/hBN} = 0.508$ nm and $d_{WSe_2/MoS_2} = 0.651$ nm giving a distance for device group A with an hBN spacer of $d_{\text{with hBN}} = 0.528$ nm+0.508 nm= 1.036 nm and for device group B without hBN spacer of $d_{\text{without hBN}} = 0.651$ nm. For samples with hBN we assume $\epsilon_{r_{\text{with hBN}}} = 4$ and for samples without hBN we assume a vdW gap, hence $\epsilon_{r_{\text{without hBN}}} = 1$. This simple estimation yields a carrier concentration of $n_{A1} \sim 2.8 \cdot 10^{12}$ cm^{-2} for device A1 and $n_{B1} \sim 8.3 \cdot 10^{11}$ cm^{-2} for device B1 at a voltage of 1.6 V. Supplementary Figure 13 presents the results for devices A1, A2 and B1, B2 as a function of bias voltage.

The Figure only depicts points based on values extracted from the measurement and no interpolation. It can clearly be seen that both devices with an hBN spacer show carrier

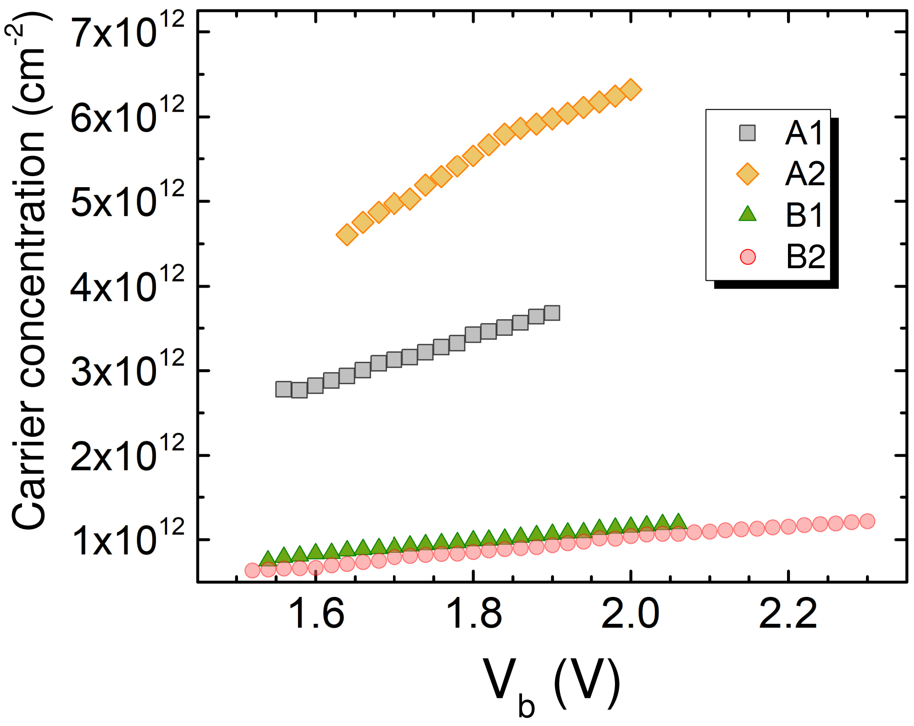

Supplementary Figure 13. Carrier concentrations as a function of bias voltage. The graph summaries the results of the estimation using the blueshift of the IX emission for devices A1, A2, B1 and B2.

concentrations in the 10^{12} cm^{-2} range whereas devices without hBN are in the range of 10^{11} cm^{-2}. Both presented methods, i.e. the MoS_2 trion shift and IX blueshift, are in good agreement and show that the charge carrier concentrations are much larger in the case of the samples with an hBN spacer.

Supplementary References

1 Wang, Z., Zhao, L., Mak, K. F. & Shan, J. Probing the spin-polarized electronic band structure in monolayer transition metal dichalcogenides by optical spectroscopy. *Nano Lett.* **17**, 740–746 (2017).

2 Mak, K. F. *et al.* Tightly bound trions in monolayer MoS_2. *Nat. Mater.* **12**, 207–211 (2013).

3 Ganchev, B., Drummond, N., Aleiner, I. & Falko, V. Three-particle complexes in two-dimensional semiconductors. *Phys. Rev. Lett.* **114**, 107401 (2015).

4 Jadczak, J. *et al.* Probing of free and localized excitons and trions in atomically thin WSe_2, WS_2, $MoSe_2$ and MoS_2 in photoluminescence and reflectivity experiments. *Nanotechnology* **28**, 395702 (2017).

5 Latini, S., Winther, K. T., Olsen, T. & Thygesen, K. S. Interlayer excitons and band alignment in MoS_2/hBN/WSe_2 van der waals heterostructures. *Nano Lett.* **17**, 938–945 (2017).
